# Identification of Natural Antisense Transcripts in Mouse Brain and Their Association With Autism Spectrum Disorder Risk Genes

**DOI:** 10.3389/fnmol.2021.624881

**Published:** 2021-02-25

**Authors:** Baran Koç, Geoffrey Fucile, Roland Schmucki, Nicolas Giroud, Tobias Bergauer, Benjamin J. Hall

**Affiliations:** ^1^Faculty of Science, University of Basel, Basel, Switzerland; ^2^Pharma Research and Early Development, Roche Innovation Center Basel, Basel, Switzerland; ^3^Neuroscience Discovery, Roche Innovation Center Basel, Basel, Switzerland; ^4^sciCORE Computing Center, University of Basel, Basel, Switzerland; ^5^Pharmaceutical Sciences, Roche Innovation Center Basel, Basel, Switzerland

**Keywords:** natural antisense transcripts, autism, ASD, lncRNA, development, mPFC, striatum, antisense transcriptome

## Abstract

Genome-wide sequencing technologies have greatly contributed to our understanding of the genetic basis of neurodevelopmental disorders such as autism spectrum disorder (ASD). Interestingly, a number of ASD-related genes express natural antisense transcripts (NATs). In some cases, these NATs have been shown to play a regulatory role in sense strand gene expression and thus contribute to brain function. However, a detailed study examining the transcriptional relationship between ASD-related genes and their NAT partners is lacking. We performed strand-specific, deep RNA sequencing to profile expression of sense and antisense reads with a focus on 100 ASD-related genes in medial prefrontal cortex (mPFC) and striatum across mouse post-natal development (P7, P14, and P56). Using *de novo* transcriptome assembly, we generated a comprehensive long non-coding RNA (lncRNA) transcriptome. We conducted BLAST analyses to compare the resultant transcripts with the human genome and identified transcripts with high sequence similarity and coverage. We assembled 32861 *de novo* antisense transcripts mapped to 12182 genes, of which 1018 are annotated by Ensembl as lncRNA. We validated the expression of a subset of selected ASD-related transcripts by PCR, including *Syngap1* and *Cntnap2*. Our analyses revealed that more than 70% (72/100) of the examined ASD-related genes have one or more expressed antisense transcripts, suggesting more ASD-related genes than previously thought could be subject to NAT-mediated regulation in mice. We found that expression levels of antisense contigs were mostly positively correlated with their cognate coding sense strand RNA transcripts across developmental age. A small fraction of the examined transcripts showed brain region specific enrichment, indicating possible circuit-specific roles. Our BLAST analyses identified 110 of 271 ASD-related *de novo* transcripts with >90% identity to the human genome at >90% coverage. These findings, which include an assembled *de novo* antisense transcriptome, contribute to the understanding of NAT regulation of ASD-related genes in mice and can guide NAT-mediated gene regulation strategies in preclinical investigations toward the ultimate goal of developing novel therapeutic targets for ASD.

## Introduction

Autism spectrum disorder (ASD) is a complex neurodevelopmental condition that manifests itself in early childhood with social interaction deficits, impaired communication and behavioral disturbances such as stereotypy and excess repetition (American Psychiatric Association, and American Psychiatric Association DSM-5 Task Force, 2013). ASD is currently estimated to affect 1 in 68 individuals (Elsabbagh et al., 2012). Disturbances in frontal cortex, amygdala and cerebellum have been associated with autism after imaging or postmortem studies of ASD patients (Amaral et al., 2008). While pathological changes in medial prefrontal cortex (mPFC) function likely contribute to impaired social behavior and communication, striatal circuit deficits likely underlie the repetitive and stereotypical behaviors (Fuccillo, 2016). Both environmental and genetic factors can contribute to ASD (Risch et al., 2014). With recent advancements in genome-wide sequencing technologies, an increasing number of protein coding gene alterations have been linked to ASD (Quesnel-Vallieres et al., 2019). However, whole genome sequencing of samples from ASD families have identified potentially disease-relevant, non-coding RNA (ncRNA) variants in the human genome (Ramaswami and Geschwind, 2018).

Non-protein coding DNA regions can be transcribed into two general ncRNA classes based on their nucleotide length: small ncRNA (<200 bp) and long ncRNAs (≥200 bp) (Elling et al., 2016; Quan et al., 2017). Long ncRNAs (lncRNAs) up to 50 kbp have been annotated so far in the human genome and a significant portion of these genes (40%) show brain-specific expression (Derrien et al., 2012; Briggs et al., 2015). Natural antisense transcripts (NATs) are a specific class of lncRNAs which are synthesized from the DNA strand opposite from protein coding genes, with which they have sequence complementarity (Katayama et al., 2005; Magistri et al., 2012). NATs can regulate the expression of their sense mRNA partners by affecting cis or trans regulatory elements (Chen et al., 2005; Velmeshev et al., 2013). Many lncRNAs have been proposed to have important roles in brain development and their dysregulation in neurodevelopmental disorders including ASD (Qureshi et al., 2010; Esteller, 2011; Qureshi and Mehler, 2013; van Devondervoort et al., 2013; Velmeshev et al., 2013; Barry, 2014; Roberts et al., 2014; Meng et al., 2015; Merelo et al., 2015; Quan et al., 2017; Cogill et al., 2018; Cuevas-Diaz Duran et al., 2019). Animal models are useful for elucidating the biological functions of NATs as well as developing and testing therapeutics that aim to modulate gene expression.

Here, we used a deep RNA sequencing approach to profile sense and antisense reads with a focus on 100 ASD-related genes in the mPFC and striatum across mouse post-natal development. The postnatal timepoints (P7, P14, and P56) we chose in this study were intended to span early postnatal cortical development, through periods of synaptic pruning and into adulthood. These timepoints have been focus in mouse brain development research allowing comparison to historical data (Thompson et al., 2014).

Using this RNA sequencing data, we built a *de novo* antisense transcriptome and then used this to identify antisense transcripts in mouse that are highly similar to the human genome. The information provided here can guide efforts to test NAT-mediated regulation of ASD-related genes.

## Materials and Methods

### Animals and Tissue Preparation

Experiments were conducted in adherence to the Swiss federal ordinance on animal protection and approved by the Canton of Basel Stadt Veterinary Authority. Wild-type mice were from C57BL/6 background and obtained from Janvier Labs (Le Genest-Saint-Isle, France). Brains were removed at three different postnatal ages (P7, P14, and P56) during daytime (12:00–16:00). There were 2, 5, and 5 animals from P7, P14, and P56 groups, respectively, with mixed biological sex (see Supplementary Table 1 for biological sex information). Following removal, brains were immediately cooled in ice-cold Hank’s balanced salt solution. Each brain hemisphere was transferred into cold RNA*later* RNA Stabilization Solution and kept at 4°C for 24 h, and transferred to −20°C until sample collection for RNA isolation.

On the day of RNA isolation, samples were thawed on ice. Each brain sample was manually sliced at room temperature into approximately 1 mm-thick coronal sections. Anatomical locations of mPFC and striatum were determined under stereo microscope according to a published protocol (Spijker, 2011). mPFC or striatum samples were collected from brain slices using a tissue punch, 2 mm diameter for P14 and P56 animals, and 1.5 mm in diameter for P7 animals. Striatal samples were a mixture of dorsal and ventral regions. For quality assessment, we used tissue annotation markers for each sample, and they were consistent with their respective tissue origins.

### RNA Sequencing Library Preparation

Total RNA including miRNA fractions were isolated using Qiagen miRNeasy mini kit according to the manufacturer’s protocol. RNA integrity was assessed with an Agilent 2100 Bioanalyzer using an Agilent Bioanalyzer 6000 Nano kit. Input of 400 ng of total RNA was used as starting material for each sample and libraries were prepared using paired-end TruSeq Stranded Total RNA LT with Ribo-Zero Gold Depletion Kit from Illumina. Quantification was performed using KAPA Library Quantification Kit and the average size of 300 bp was determined by using a High Sensitivity DNA Kit. The libraries were pooled and diluted to 13 pM to load on an Illumina HiSeq 2500 Instrument. Read lengths were 50 bp, and each sample was sequenced to depths of between ∼135–185 million reads with an average depth of ∼162 million reads.

### *De novo* Antisense Transcriptome Assembly and Differential Expression Analysis

**FIGURE 1:**
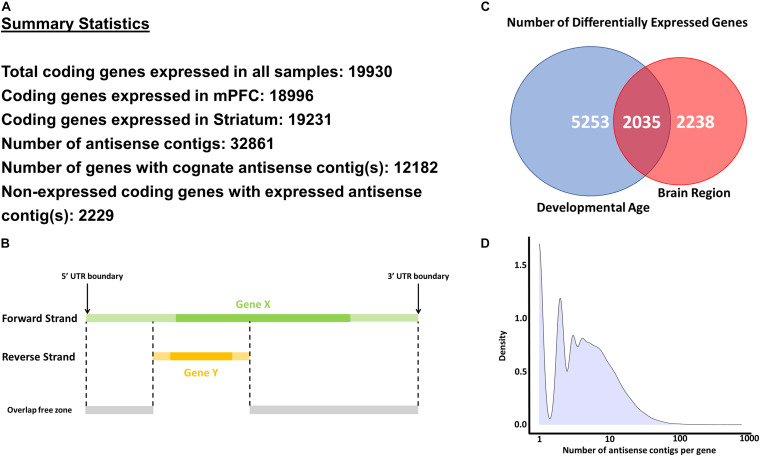
Methodology and key statistics for the current study. **(A)** Summary statistics for the main analyses conducted in the study. **(B)** A schematic explaining the methodology to extract antisense reads for a selected gene (Gene X). Only antisense reads in the overlap free zone were used in analyses. **(C)** Number of differentially expressed genes across developmental age and brain regions. **(D)** Histogram of number of antisense contigs per gene.

Reads which mapped in antisense orientation to genomic features (i.e., genes) were used to create a *de novo* antisense transcriptome. Reads were first aligned against the Ensembl mouse genome reference GRCm38, including all annotated splice junctions, using STAR v2.7.3a (Dobin et al., 2013). A table of all known and novel splice junctions is provided in Supplementary File 1 (see Splice Junctions sheet). On a per-gene basis, SAMtools v1.7 (Li et al., 2009) was used to extract reads which aligned as antisense within UTR boundaries (Figure 1B). *De novo* transcriptome assembly using the collection of antisense reads from all samples was performed using the Trinity platform v2.8.4 using library strand information (Grabherr et al., 2011; Haas et al., 2013) (see Data Availability Statement). The Trinity contigs were first filtered by BLAST analysis (99% identity and ≥95% query coverage) against the GRCm38 whole-genome reference (see Blast Analysis of Contigs Sheet in Supplementary File 1). These contigs were added to the GRCm38 reference annotations with all known lncRNA and antisense features removed, and a new index for read mapping using STAR was generated. Alignment of all samples against the STAR index containing GRCm38 and filtered Trinity contig annotations was then performed, and uniquely mapped read counts were extracted using the stranded counting methods of STAR. The use of uniquely mapped reads for counting eliminated spurious contigs from overlapping features with opposite strandedness. To remove potentially spurious antisense contigs due to reported strand misassignment rates of up to 3% (Levin et al., 2010; Zeng and Mortazavi, 2012; Licht et al., 2019), we further filtered the antisense contigs to those with aligned reads totaling more than 3% of the combined reads of the sense strand of the gene to which the contig is mapped plus the reads aligned to the antisense contig itself (see Contigs – Filtered_Detected sheet in Supplementary File 1). This low-pass filter and use of uniquely mapped criterion for read counting eliminated spurious contigs from overlapping features with opposite strandedness. The STAR index and mapping was recomputed using the BLAST-validated and low-count filtered Trinity contig annotations combined with GRCm38 protein coding feature annotations in the GTF format (see Data Availability Statement) for the final round of mapping and read counting. The segments of contigs discontinuously mapped within gene bodies are modeled as separate exons in the GTF annotations. Figure 1 indicates key summary statistics regarding the number of expressed genes and antisense contigs.

### Differential Expression Analysis

Differential expression analysis was conducted using DESeq2 v1.24.0 (Love et al., 2014) using the Wald test, based on read count matrices derived from uniquely mapped reads from the STAR alignments. Genes expressed in their natural sense strand were filtered to include those with at least 10 uniquely mapped reads in at least three different samples. For principal components analysis (computed with the prcomp function in R v3.6.0) and hierarchical clustering analysis (computed in R v3.6.0 using Pearson distance and Ward D2 agglomeration), counts were normalized using the variance stabilizing transform as implemented in DESeq2. Functional enrichments were computed using clusterProfiler (Yu et al., 2012; Jassal et al., 2020) and ReactomePA (Yu and He, 2016). The raw read count matrices for the coding gene expression, antisense contig expression, and the contigs mapped to known lncRNAs are provided in Supplementary File 2.

### Enrichment of Antisense Expression Across Brain Regions

We developed a simple formula that demonstrates the relative abundance of antisense reads across two brain regions, mPFC and striatum. Where a positive tissue specificity score (TSS) specifies greater abundance of mPFC reads, and a negative TSS would indicate increased relative abundance in striatum. Total Reads relate to the counts on the coding and opposite strands while Opp_Reads relate to the counts only on the opposite strand. mPFC relates to counts in mPFC, Str refers to counts in Striatum, and Total relates to all the counts from mPFC and Striatum. TSS is calculated for each gene separately using counts normalized by library size.

$$\text{Tissue specificity score}=100\times \frac{\left(\frac{mPFC_{Opp_{Reads}}}{mPFC_{Total}}\right)-\left(\frac{Str_{Opp_{Reads}}}{Str_{Total}}\right)}{\left(\frac{mPFC_{opp_{Reads}}+Str_{Opp_{Reads}}}{TotalReads}\right)}$$

### Identifying Antisense Reads for ASD-Related Genes

We adopted a list of 103 ASD-related genes from a previously published study which used a bioinformatics pipeline to identify NATs in genomic regions related to ASD (Betancur, 2011; Velmeshev et al., 2013). We screened mouse orthologues of these genes on Ensembl (Release 90) and found that 100 of these genes have a 1-1 mouse orthologue based on Ensembl annotations (*NLGN4X*, *ZNF674*, and *ZNF81* were the exceptions) (see Supplementary File 3 for complete gene list). We used these 100 ASD-related genes in our analyses unless otherwise stated.

### Linear Modeling to Compare Sense and Antisense Expression

Linear models were implemented in R v3.6.0 using the lm function. Models were implemented per gene or antisense contig within mPFC and striatum samples separately using all replicates, and considered significant with *p* < 0.05 for the model *F*-test. Models were first computed using P7, P14, and P56 samples, and in the case of failed F-test, a linear model fit was attempted using only P14 and P56 samples. The single parameter estimate (slope) was then compared between sense and antisense partners.

### Determination of Putative NATs With High Similarity to Human Genome

Putative NATs from mouse which mapped as antisense to the human genome (GRCh38) were determined by BLAST analysis. The criteria for high similarity between mouse and human were set as >90% coverage and >90% identity.

### PCR for Assessing Putative NAT Expression

PCR was performed using AgPath-ID One-Step RT-PCR kit (Cat# 4387391, Thermo Fisher Scientific) according to the recommended protocol with a Roche LightCycler 480 Instrument (Roche Molecular Systems, Inc., Pleasanton, CA, United States). Pre-designed and custom-designed TaqMan probes (Thermo Fisher Scientific) were used (see Supplementary File 4 for TaqMan probe sequences). *C*_*t*_ values were calculated using absolute quantification/2nd derivative maximum method in high confidence mode with LightCycler 480 Software, Version 1.5. Probes were designed against antisense transcripts mapped to *Mef2c* (TRINITY_DN4422_c0_g5_i1), *Crebbp* (TRINITY_DN76675_c0_g1_i1), *Cacna1c* (TRINITY_ DN47072_c0_g1_i1), *Rpgrip1l* (TRINITY_DN47110_c0_g1_i1), *Foxp1* (TRINITY_DN19745_c0_g1_i1), *Cntnap2* (TRINITY_ DN86307_c0_g1_i1), *Syngap1* (TRINITY_DN24116_c0_g2_i1, TRINITY_DN33950_c1_g3_i1), and *Prss12* (TRINITY_DN2 5745_c0_g2_i1) (Supplementary File 4).

## Results

### Profiling ASD-Related Genes in mPFC and Striatum Across Development Revealed Differentially Expressed Genes

**FIGURE 2:**
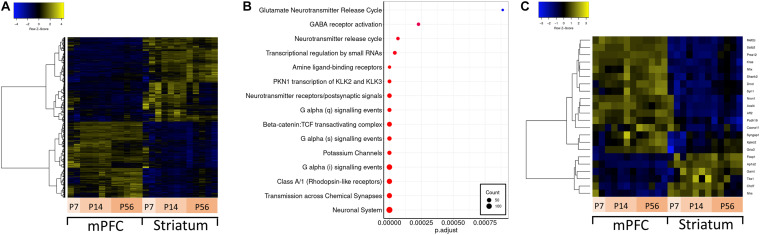
Differential expression between mPFC and striatum and over-representation analysis. **(A)** Hierarchically clustered heatmap of all genes differentially expressed in mPFC versus striatum. **(B)** Dotplot of top enriched Reactome pathway annotations related to the central nervous system among genes differentially expressed in mPFC versus striatum. **(C)** Hierarchically clustered heatmap of ASD-related genes differentially expressed in mPFC versus striatum.

Brain region-specific expression of genes can give insight about their function in disease relevant brain regions. Thus, we first aimed to identify genes preferentially expressed in either mPFC or striatum –two brain regions implicated in ASD. For this analysis, we conducted differential expression analysis contrasting all mPFC versus striatum samples (Figure 2A). Out of 19930 expressed genes, there are approximately 4300 genes which are significantly differentially expressed between mPFC and striatum. Among these, there is an enrichment of several Reactome pathways involving neuronal signaling (Figure 2B). A subset of the ASD-related genes are differentially expressed between mPFC and striatum: *Mef2c*, *Satb2*, *Prss12*, *Kras*, *Nfix*, *Shank2*, *Dmd*, *Syn1*, *Nrxn1*, *Acsl4*, *Aff2*, *Pcdh19*, *Cacna1f*, *Syngap1*, *Iqsec2*, and *Gria3* are enriched in mPFC; *Foxp1*, *Ap1s2*, *Gamt*, *Tbx1*, *Chd7*, and *Nhs* are enriched in striatum (Figure 2C).

**FIGURE 3:**
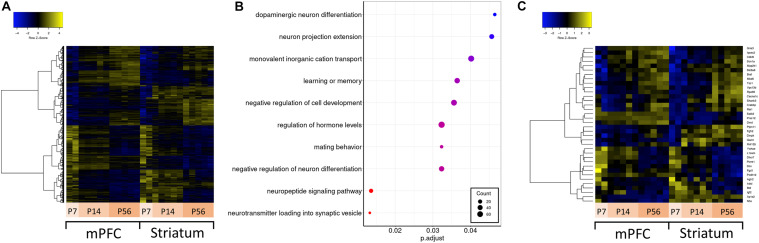
Differential expression across development and over-representation analysis. **(A)** Hierarchically clustered heatmap of all genes differentially expressed across developmental ages. **(B)** Dotplot of top enriched Gene Ontology annotations related to the central nervous system among genes differentially expressed across developmental ages in mPFC. **(C)** Hierarchically clustered heatmap of ASD-related genes differentially expressed across developmental ages.

Autism spectrum disorder is a neurodevelopmental disorder; therefore, understanding how ASD-related genes are regulated over development is important for understanding their function in synaptic development and circuit formation. To understand their developmental regulation, we conducted differential expression analysis across developmental stages (P7, P14, and P56) for both mPFC and striatum samples separately, and compared the overlap and expression profile of differentially expressed genes (Figure 3A). These developmental time points overlap with transcriptomic changes related to synaptogenesis and synaptic maturation in mouse (Dillman and Cookson, 2014) and correspond to various stages of human brain development spanning from childhood to mature adult (Christakis et al., 2018). There are 19231 genes expressed in striatum, and 18996 in mPFC. Among these detected genes, there are 7288 genes which vary across development in either mPFC or striatum, of which 3390 are differentially expressed in both mPFC and striatum (1979 are mPFC-specific, and 1919 are striatum-specific). The mPFC-specific genes are enriched for regulation of cognitive function and neuronal signaling and development (Figure 3B), whereas no significant enrichment of functional terms or pathways was detected for the striatum-specific genes. Among the ASD-related genes, 36 show a developmentally regulated expression profile (Figure 3C).

In order to rule out any bias in gene expression due to use of mixed sex of animals, we performed differential expression analysis and found that 21 genes showed differences between sexes considering all three time points (P7, P14, and P56) (see Supplementary Table 2). A significant portion of the differentially expressed genes (e.g., *Eif2s3x/y*, *Dby/Ddx3y*, *Smcy/Kdm5d*, *Uba1y/Ube1y*, *Uty*) overlapped with a previously published study where sex differences in sex chromosome gene expressions were determined in the mouse brain (Xu et al., 2002).

### A *de novo* Transcriptome Assembly Approach Identified Putative ASD-Related NATs

A detailed antisense transcriptome is lacking for the mouse genome with mPFC and striatum specific annotations. To fulfill this need, we used deep RNA sequencing to assemble an antisense RNA transcriptome from mouse mPFC and striatum samples using reads mapped against the antisense strand of coding genes for the entire GRCm38 genome. We used a bioinformatics approach for verifying the resultant contigs after assembly by BLAST analysis against the mouse genome and a low-pass filter of uniquely mapped read counts. Our verification approach reduced 143811 contigs to 32861, mapped to 12182 genes (see Supplementary Materials and Data Availability Statement). The length distribution for these contigs is centered around 250 bp with a long flat tail up to ∼20 kbp (see Supplementary Figure 1). Using a BLAST analysis with a cutoff of >95% query coverage and 99% identity, 2239 of these contigs were mapped to 1018 known lncRNA sequences from the GRCm38 annotations (see Supplementary File 5). These antisense contigs mapped to known lncRNA also show dynamic expression across tissues and development (see Supplementary Figure 2).

**FIGURE 4:**
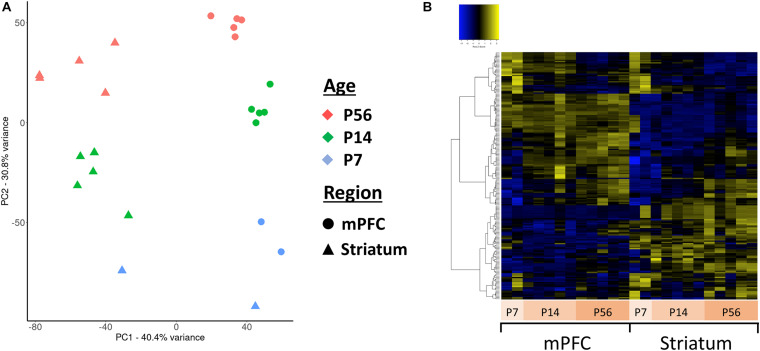
Principal component analysis (PCA) and clustering of the assembled contigs. **(A)** Plot of the first two principal components for the normalized expression values of all antisense contigs. **(B)** Hierarchically clustered heatmap of contigs mapped to ASD-related genes expressed across developmental ages and between mPFC and striatum.

Principal component analysis (PCA) of the assembled contigs revealed a clear clustering based on brain region (niche) and developmental stage (age) (Figure 4A), but not biological sex (Supplementary Figure 3). This clearly demonstrates that the assembled *de novo* contigs of antisense reads are sufficient to distinguish samples based on their tissue origin and age. Essentially the same PCA clustering is observed using the coding gene expression matrix. We next focused our analyses to the 271 antisense contigs which mapped to ASD-related genes. We identified 140 differentially expressed antisense contigs between mPFC and striatum, and 76 that were differentially expressed across developmental time (Figure 4B).

### Subset of Antisense Contigs Showed Differential Expression in mPFC or Striatum

To identify which genes show the most antisense expression, we compared the sum of normalized counts from every sample for each gene against the sum of all normalized counts for each cognate antisense contig. With respect to the 100 ASD-related genes, *Fgfr2* showed the highest ratio of antisense to total reads (see Supplementary File 6). According to the Aceview Transcriptome Database (Thierry-Mieg and Thierry-Mieg, 2006), the genes with reported macaque and human NATs and antisense expression in mouse are *Braf*, *Cacna1c*, *Foxp1*, *Nf1*, *Pafah1b1*, *Ube3a*, *Vps13b* (Supplementary File 3). These genes show an antisense:total reads ratio between 0.12 and 0.45 in our analysis.

**FIGURE 5:**
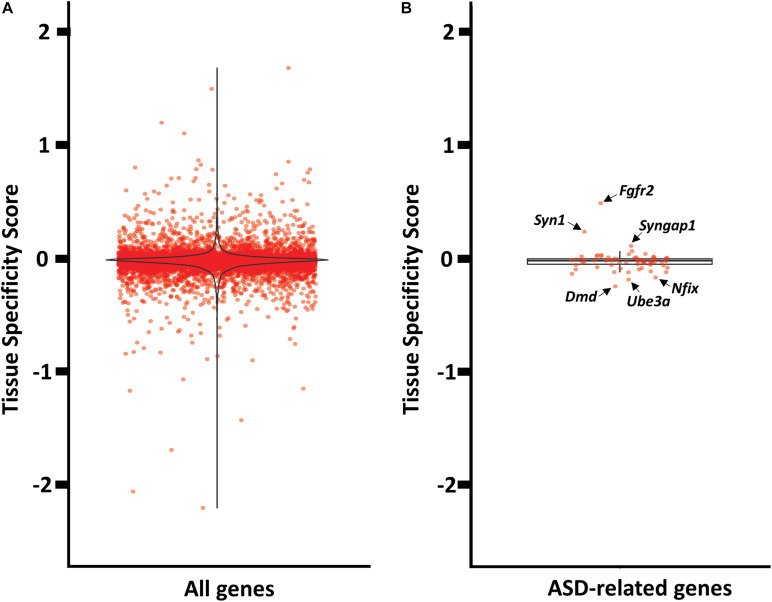
Tissue enrichment analysis of genes with at least one cognate antisense contig. **(A)** Violin plot of tissue specificity score distribution for all genes with at least one cognate antisense contig. **(B)** Box plot of tissue specificity score distribution for ASD-related genes with at least one cognate antisense contig.

Next, we quantified the relative abundance of antisense expression per gene in mPFC versus striatum, which might suggest a brain specific regulation. We used a simple measure (tissue specificity score) of the proportion of antisense reads in mPFC and striatum (see Supplementary File 7 for tissue_specificity_scores). Our analyses revealed that only a fraction of antisense reads displayed mPFC or striatum enrichment (Figure 5A). The five genes with the most mPFC-enriched antisense expression are *Itgb6*, *Gm20696*, *Pif1*, *Dnah14*, and *Espn*, while the five top genes with striatum-enriched antisense expression are *Cd6*, *Acot5*, *Gas7*, *S1c15a5*, and *Tmprss4*. Among the 72 ASD-related genes with antisense expression, none appear to be highly enriched in either mPFC or striatum. The mPFC-enriched outliers are *Fgfr2*, *Syn1*, *Syngap1*, and *Satb2*, and striatum-enriched outliers include *Dmd*, *Ube3a*, *Nfix*, and *Dmpk* (Figure 5B).

### Expression of Antisense Contigs and Their Cognate Genes Across Developmental Time Displayed Primarily Positive Relationships

**FIGURE 6:**
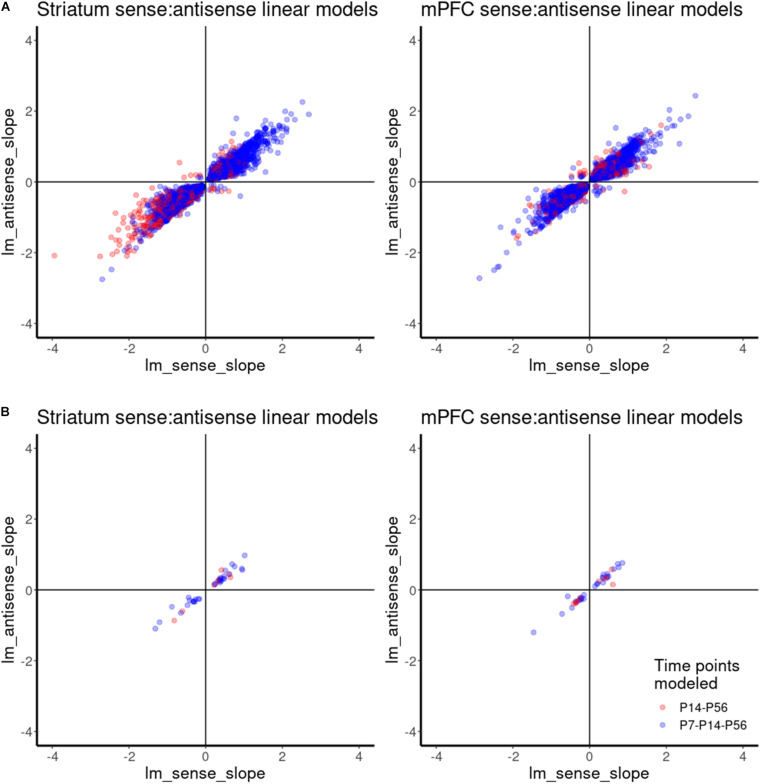
Correlation analysis of gene-antisense contig pairs using linear model. **(A)** Scatterplot showing the relationships between linear model slopes for all gene-antisense contig pairs. **(B)** Scatterplot showing the relationships between linear model slopes for ASD-related gene-antisense contig pairs.

Similar to differential spatial expression, temporal correlation of sense and antisense reads through development could also indicate a possible regulatory, functional mechanism for antisense reads. Therefore, we investigated the correlation between antisense contigs and their cognate gene. Given the small number of samples and classes for this study, we compared the slopes of linear models for the abundance estimates of antisense contigs and their cognate genes across developmental time as a proxy for correlation, rather than Pearson correlation using means from replicates. This allowed us to incorporate the variance estimates from replicates. A linear model accurately captured the expression dynamics of 6185 antisense contigs and cognate gene pairs in mPFC, and 5733 antisense contigs and cognate gene pairs in striatum. We infer a positive correlation when the slopes of expression across developmental age of an antisense contig and its cognate gene are both positive or both are negative, as observed in the upper right and lower left quadrants of Figure 6. A negative correlation is inferred when an antisense contig and its cognate gene have mismatched positive and negative slopes, as observed in the upper left and lower right quadrants of Figure 6. This analysis indicates that there is a positive correlation in expression between the vast majority of antisense contigs and cognate gene pairs (Figure 6A). There were 30 and 22 cases of negative correlation in mPFC and striatum, respectively There were 38 and 41 gene-antisense contig pairs among ASD-related genes for which a linear model could be fit accurately for mPFC and striatum, respectively, all of which had a positive relationship between slopes (Figure 6B). The expression dynamics of *Syngap1* was not accurately captured by a linear model. However, we do observe that in contrast to the findings of Velmeshev et al., 2013, there does not appear to be significant negative correlation of *Syngap1* and any cognate antisense contigs (Supplementary Figure 6). Rather, it appears that *Syngap1* expression is positively correlated with its cognate antisense contigs (Supplementary Figures 6A,B). However, we do observe a consistent enrichment of *Syngap1* in mPFC (Figure 5B).

### A Subset of ASD-Related Contigs Is Highly Similar to Human

The conservation of antisense transcripts between mouse and human supports a conserved functional role. We conducted a BLASTn analysis of the antisense contigs identified from the mPFC and striatum transcriptomes against the human genome. Our analysis identified 110 antisense contigs mapping to 68 of our short list of ASD-related genes with >90% coverage and >90% identity to their respective human gene locus (see Supplementary File 8). To further validate these putative NATs, we checked for their expression using previously published human brain transcriptomes. RNA-seq data of the prefrontal cortex right hemisphere from a 72 year old male diagnosed with Parkinson’s disease (Li et al., 2020) (GEO Sample GSM3984160) expressed 79 of the 110 ASD-related antisense contigs conserved between human and mouse with at least 50 uniquely mapped reads and a mean of 230 reads. By contrast, the average number of uniquely mapped reads among all antisense contigs for this brain sample was ∼50. Similarly, the dorsal lateral prefrontal cortex (Brodmann Area 9) of a male non-psychiatric control (Pantazatos et al., 2017) (GEO Sample GSM2705369) expressed 83 of the 110 conserved ASD-related antisense contigs, with a mean of 294 reads compared to 45 reads for all antisense contigs.

### Expression Validation of *de novo* Antisense Contigs as Putative ASD-Related NATs

Next, we selected some of the highly conserved and non-conserved ASD-related antisense contigs for PCR verification (see Supplementary File 8 and Supplementary Figure 4). We used a combination of criteria for selecting non-conserved contigs such as predicted antisense expression in human (*Syngap1*, *Cacna1c*, and *Cntnap2*) and suggestive evidence for a regulatory role in the mouse brain. We designed custom TaqMan probes for the selected transcripts (see Supplementary File 4 for list of selected transcripts and TaqMan probe sequences) and validated their presence by PCR using total RNA isolated from mouse brain tissue (Supplementary Figure 4). We confirmed the size of the expected PCR-products by agarose gel electrophoresis (Supplementary Figure 4) except for one sample. Given that we could successfully confirm the presence of all selected *de novo* contigs by PCR, we consider them as putative NATs but their functional significance awaits further testing.

## Discussion

Here, we investigated the differential expression of 100 ASD-related genes and their antisense partners in mouse using a deep RNA sequencing approach (>130 million reads/sample). Our comprehensive study demonstrates that ASD-related antisense transcripts are differentially regulated in two ASD-associated brain tissues (mPFC and striatum) through brain development. To the best of our knowledge, our study is unique for dissecting NAT expression at such resolution and providing brain region specific and developmental information. Moreover, we assembled a *de novo* antisense transcriptome yielding 32861 contigs that were verified by BLAST analysis, and a low read count filter, identified a subset with high similarity to the human genome. As further validation, we mapped 2239 contigs to 1018 known lncRNAs. This suggests that although we are identifying previously described lncRNA, our sequencing depth may be insufficient in some cases for full reconstruction. By extension it is likely many of our 32861 antisense contigs could be further collapsed with increased sequencing depth. However, we have clearly identified a wealth of novel antisense expression phenomena. Our *de novo* antisense transcriptome can be used as a point of reference for selecting NAT candidates in CNS research.

The 100 genes we used in this study do not represent all ASD-related genes. For example, 1003 human genes implicated in autism are listed by the SFARI gene database in the 2020 Q4 release^1^ and in other published studies (Sanders et al., 2015; Iakoucheva et al., 2019). However, our list provides a good one-to-one comparison with the study where expression of human NATs were examined for the same ASD-associated genomic regions in human (Velmeshev et al., 2013). For example, *SYNGAP1*-antisense was shown to be differentially expressed in autistic brains compared to the brains of control subjects. No *Syngap1* antisense transcript has been reported so far in mice or rats; therefore, using animal models to study NAT-mediated gene regulation of *Syngap1* has not been possible. In our *de novo* antisense transcriptome, we could assemble several antisense transcripts that are related to the *Syngap1* locus (Supplementary Figures 6A,B) and validated the expression of two of these in mouse brain tissue by PCR (Supplementary Figure 4). If regulatory functions of these transcripts can be demonstrated, they would serve as a novel modality to regulate *Syngap1* expression in mouse models similar to *Ube3a* (Meng et al., 2015), *Bdnf* (Modarresi et al., 2012), and *Bace1* (Faghihi et al., 2008) among others.

Our assembled antisense contigs exclude overlapping genomic elements that were present on the reverse strand of protein coding genes to eliminate their contribution to abundance estimates. We are not aware of any example where a protein-coding gene can act as an antisense transcript to regulate the expression of another protein-coding gene in the reverse strand. In case such regulation is present, we might have biased our search and excluded protein-coding genes with antisense function.

Brain region-enriched expression can pinpoint a specific function for the investigated gene or transcript. This is mostly likely due to the result of the regions having a different cell type composition for executing their unique function within their microcircuitry. For example, *Foxp1* is a good example of an autism-related gene with a demonstrated striatum-specific function (Araujo et al., 2015). Foxp1 regulates excitability of medium spiny neurons in striatum and its reduction was correlated with ultrasonic vocalization deficits in mouse. In parallel to this finding, our analysis demonstrated that *Foxp1* and several cognate antisense contigs were highly enriched in striatum (Figure 2C). Another noteworthy example is *Prss12* (aka motopsin) gene, which showed strong enrichment in mPFC (Figure 2C) a relatively high antisense:total reads ratio of 0.40, and at least one cognate antisense contig also enriched in mPFC. Interestingly, *Prss12* knockout mice that were exposed to maternal separation paradigm showed decreased Cfos positive cells in the prefrontal cortex after three-chamber social test compared to wild-type controls, suggesting Prss12 activity in the prefrontal cortex is involved in emotional response (Hidaka et al., 2018). Based on our and others’ findings, and previous reports of stabilizing effects of lncRNA on cognate mRNA (Wahlestedt, 2013; Khorkova et al., 2014; He et al., 2019), we speculate that *Prss12* antisense transcript might contribute to the regulation of Prss12 function in prefrontal circuits for social behavior.

Similarly, developmental regulation of genes can also indicate specific functions affecting brain development. A good example for an ASD-related gene that is known to be developmentally regulated is doublecortin (*Dcx*) (Francis et al., 1999; Vourc’h et al., 2002). Mutations in *Dcx* cause neuronal migration deficits and are associated with mental retardation (Reiner et al., 2006). As expected, *Dcx* stood out as one of the most developmentally regulated genes in our analysis. Its expression was significantly downregulated from P7 to P56 (Figure 3C), a pattern which was also observed for one of its cognate antisense transcripts (TRINITY_DN54169_c0_g1_i1).

Expression correlation of sense and antisense partners might indicate a functional relationship. Several studies have tried to understand the link between sense-antisense transcript pairs by analyzing their correlation relationship (Khorkova et al., 2014). For example, a positive correlation was reported for sense-antisense partners that show tissue-specific expression profiles with overlapping promoter sequences (Uesaka et al., 2014). On the other hand, a negative correlation was identified for antisense transcripts that position in the introns or downstream of their protein-coding partners (Batagov et al., 2013). In our study, we observed primarily positive relationships for the temporal correlation of sense and antisense expression (Figure 6A).

For example, *Ube3a* is a known gene with antisense-mediated expression regulation (Meng et al., 2015) and it showed a relatively high antisense:total reads ratio of 0.38 (Table 1). The expression of *Ube3a* antisense contigs appears to be positively correlated with *Ube3a* expression, with the exception of one antisense contig in mPFC which appears to be negatively correlated (TRINITY_DN4415_c0_g1_i1) (Supplementary Figures 6C,D). *Scn1a*, a gene known to have an antisense gene in mouse (Hsiao et al., 2016) and in our analysis had an antisense:total reads ratio of 0.30, showed a positive correlation with several cognate antisense contigs (Supplementary Figures 6E,F). It was unexpected to observe such a large imbalance of positive correlation among sense and antisense pairs. However, it is known that antisense lncRNA plays an important role in stabilizing mRNA and facilitating transcript splicing (Wahlestedt, 2013; Khorkova et al., 2014; He et al., 2019). Transcriptional interference via lncRNA, which involves negative antisense-sense correlation, has been primarily observed in promoter regions (Kornienko et al., 2013), which was not assessed in our dataset. It is also possible these classes of lncRNA are expressed at lower levels than those with a positive antisense-sense correlation. In our study, we uniquely mapped approximately 100 million reads per sample, which may be insufficient to capture the expression of many lncRNAs which involve negative regulation of cognate mRNA. We cannot speculate with our dataset for any mechanism attributable to temporal correlation of sense and antisense partners in the absence of careful analyses conducted on a per-gene basis.

**TABLE 1:** Twenty ASD-related genes with highest ratio of antisense: total reads.

Gene	Antisense: total reads
*Fgfr2*	0.76
*Gamt*	0.50
*Hras*	0.50
*Smc1a*	0.46
*Ahi1*	0.46
*Braf*	0.45
*Kras*	0.45
*Ap1s2*	0.43
*L1cam*	0.43
*Mid1*	0.41
*Pten*	0.41
*Ywhae*	0.40
*Atrx*	0.39
*Nrxn1*	0.39
*Syngap1*	0.39
*Ube3a*	0.38
*Mef2c*	0.38
*Foxp1*	0.38
*Ptpn11*	0.38
*Cask*	0.37

Our *de novo* antisense transcriptome serves as a useful resource with 32861 assembled and detected contigs mapped to 12182 genes. This large number of antisense transcripts is consistent with previous estimates (Ling et al., 2013). The PCA of assembled transcripts resulted in a clear clustering of samples based on their brain region (niche) and development (age) (Figure 4A). Our differential expression analysis also revealed many of these contigs were significantly tissue and age specific (Figure 4B). Given that ncRNA expression can be exceptionally specific to cell types, neuroanatomical regions and subcellular compartments (Mercer et al., 2008), our findings are also consistent with the idea that lncRNAs play functional role in CNS development and diseases (Cuevas-Diaz Duran et al., 2019).

Through the use of a stranded library and uniquely mapped reads within gene bodies with a low read count filter, which reduced the number of contigs from 143811 total to 32861, we are confident that the majority of these contigs are actual antisense transcripts present in the brain. Successful validation of all nine selected contigs by PCR in the mouse brain (Supplementary Figure 4) provides further validation. Nevertheless, validation of these contigs as functional transcripts is necessary. For example, cell based, large-scale RNA interference-mediated loss of function assays (Faghihi et al., 2010) can be used to probe the biological functions of the assembled transcripts.

Conserved sequences of antisense transcripts between mice and humans could indicate a shared mechanism and facilitates experimental efforts to understand the functionality of antisense transcripts using animal models. Using BLAST analyses of our assembled novel *de novo* antisense transcripts against the human genome, we could identify highly similar sequences between two species for ASD-related genes (Supplementary File 8). *Mef2c* is particularly interesting as it has several contigs that are highly similar to the human locus, and two of which are differentially expressed (Figure 4B). Multiple non-coding and antisense RNAs have been identified around human *MEF2C* gene (Mitchell et al., 2017); however, no antisense transcript has been reported for the mouse homolog. Other genes which have conserved antisense contigs between human and mouse and display interesting differential expression include *Syngap1*, *Nrxn1*, *Syn1*, *Foxp1*, *L1cam*, *Cask*, and *Scn1a* (see Supplementary Figure 5 for detailed list of the contigs). Hence, investigating antisense regulation of these genes in mouse would be noteworthy. Nevertheless, it is also important to note that sequence homology is not the only parameter that determines similar functions in different species (Mathews et al., 2010). For instance, the brain-derived neurotrophic factor gene (*Bdnf*) in mouse and human are regulated by their respective *Bdnf*-antisense transcripts despite lack of conserved antisense sequences between two species (Modarresi et al., 2012). Therefore, a more inclusive approach should be considered when antisense transcripts are compared between two or more species.

## Limitations

In this study, the BLAST analysis of *de novo* antisense transcripts identified highly complementary and covered sequences between mouse and human. This finding is particularly important for generating preclinical models to study lncRNA function in relation to CNS diseases. However, it should be noted that sequence similarity is not the only factor that determines functional similarity in different species and a more comprehensive approach is necessary to draw conclusions (Mathews et al., 2010).

Regarding conservation of the putative NATs, the putative NATs map primarily to mRNA exons and this confounds the interpretation of what is driving the sequence conservation.

The inability to model non-linear relationships for many sense and cognate antisense transcripts indicates that the correlation analysis is incomplete, with more complex relationships to be explored in future for specific pairs of genes and cognate antisense transcripts.

The lack of additional replicates at early time points (P7) likely limited our ability to detect statistically significant developmental dynamics of sense and antisense expression. It should be also noted that this study is not powerful enough to resolve the sex related differences due to limited number of animals of different sexes. Nevertheless, sex-specific differences are not primary drivers of the transcriptome in our analysis, as evidenced by the correspondence of axes of development and brain region with the first two principal components, which capture the majority of the variance, and subsequent principal components capturing no more than ∼5% of the variance (Figure 4A).

Given that our study is contributing to the understanding of lncRNA research, epidemiological studies carried out on patients and further development in bioinformatics are crucial to have a better understanding of the lncRNA function in general and to gain a better insight into their roles in ASD and other neurodevelopmental diseases (Guo et al., 2016; Luo et al., 2017; Tang et al., 2017).

## Conclusion

The data presented here provide evidence that some ASD-related genes and their antisense transcripts are differentially expressed between mPFC and striatum through development. These differences should be taken into account to obtain a more complete view of the interplay of sense-antisense partners that lead to the disease state. Moreover, we successfully assembled *de novo* antisense transcriptome with 32861 contigs for mouse brain with tissue specific annotations. Our antisense transcriptome can be used as a reference for determining NAT candidates for research activities in the CNS and its disorders. Our bioinformatics approach to verify and mask contigs provides a more refined list of transcripts and it can be applicable to other *de novo* transcriptome assembly studies. Identifying and understanding specific antisense transcripts regulating the expression of ASD-related genes would be important to develop novel RNA-based therapeutics (Khorkova and Wahlestedt, 2017) for ASD.

## Data Availability Statement

The datasets presented in this study can be found in online repositories. The names of the repository and accession number can be found at https://www.ncbi.nlm.nih.gov/geo/query/acc.cgi?acc=GSE146628.

## Ethics Statement

The animal study was reviewed and approved by experiments were conducted in adherence to the Swiss Federal Ordinance on Animal Protection and approved by the Canton of Basel-Stadt Veterinary Authority.

## Author Contributions

BK, GF, RS, TB, and BJH conceived and designed the experiments. BK performed all wet lab experiments except for RNA-Seq which was done by NG. BK, GF, and RS analyzed the data. BK, GF, and BJH drafted and revised the manuscript. All authors contributed to the article and approved the submitted version.

## Conflict of Interest

BK, RS, NG, TB, and BJH were full time employees of F. Hoffmann-La Roche Ltd. of Basel, Switzerland during the course of studies. The remaining author declares that the research was conducted in the absence of any commercial or financial relationships that could be construed as a potential conflict of interest.

## Footnotes

1 https://gene.sfari.org/database/human-gene/
