# Supplementary figures and images for: Identification of Natural Antisense Transcripts in Mouse Brain and Their Association With Autism Spectrum Disorder Risk Genes
Source: Front Mol Neurosci. 2021 Feb 25;14:624881. doi: 10.3389/fnmol.2021.624881 (PMC7947803; doi:10.3389/fnmol.2021.624881)

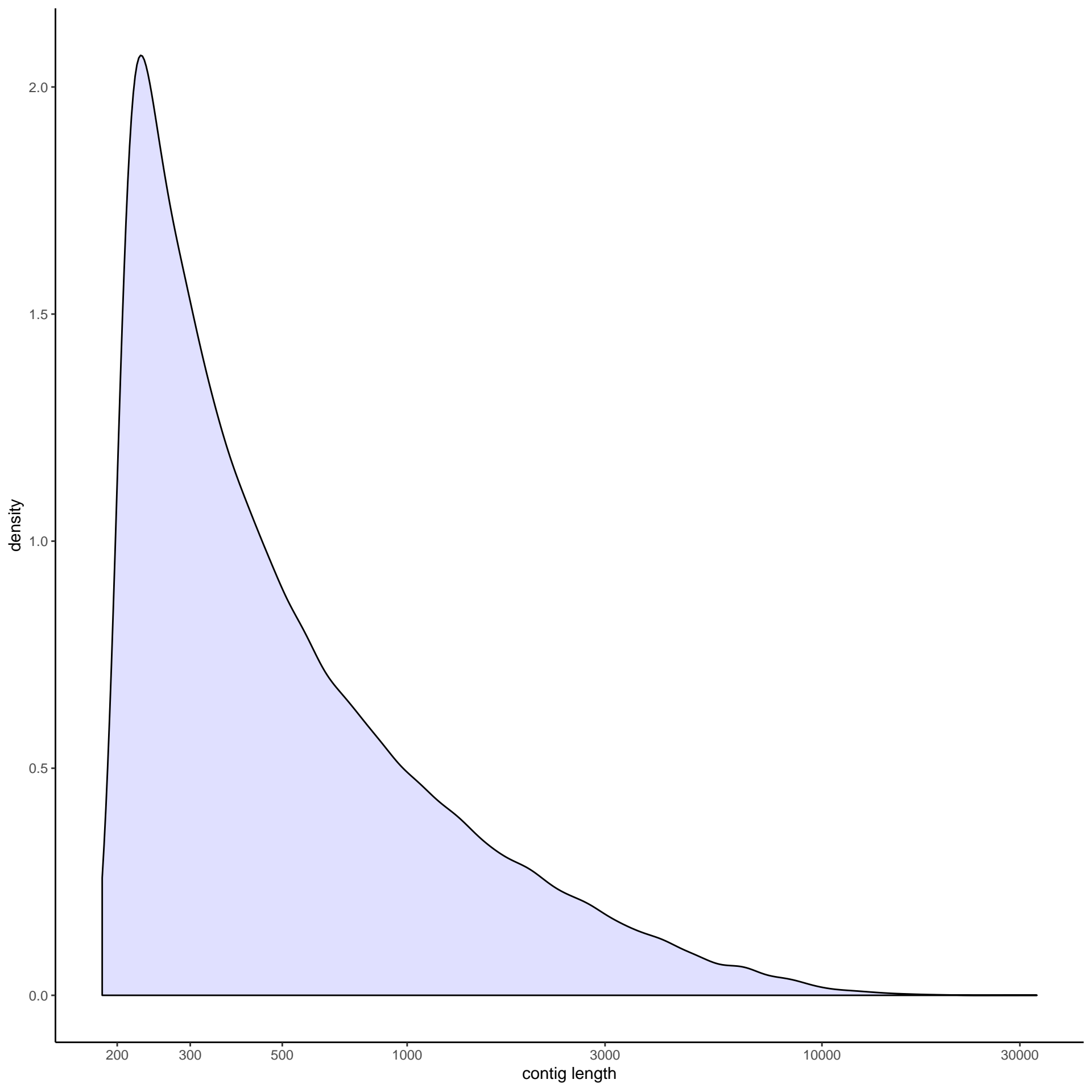

Supplement: Supplementary file 1 [file Image_1.PDF]

configs mapped to known lncRNA

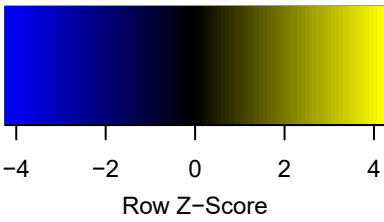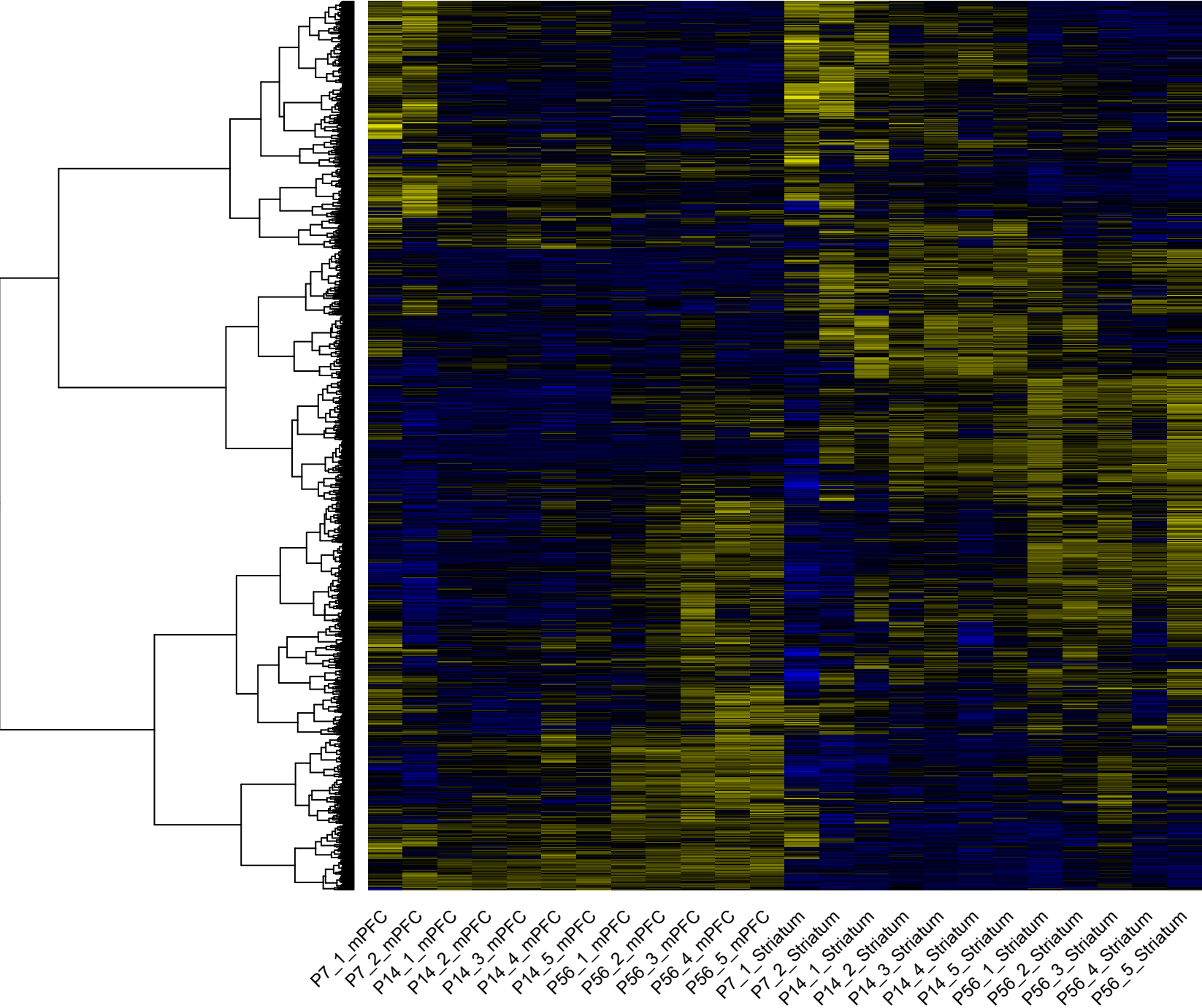

Supplement: Supplementary file 2 [file Image_2.PDF]

PCA of VST-normalized gene counts (GRCm38 annotations)

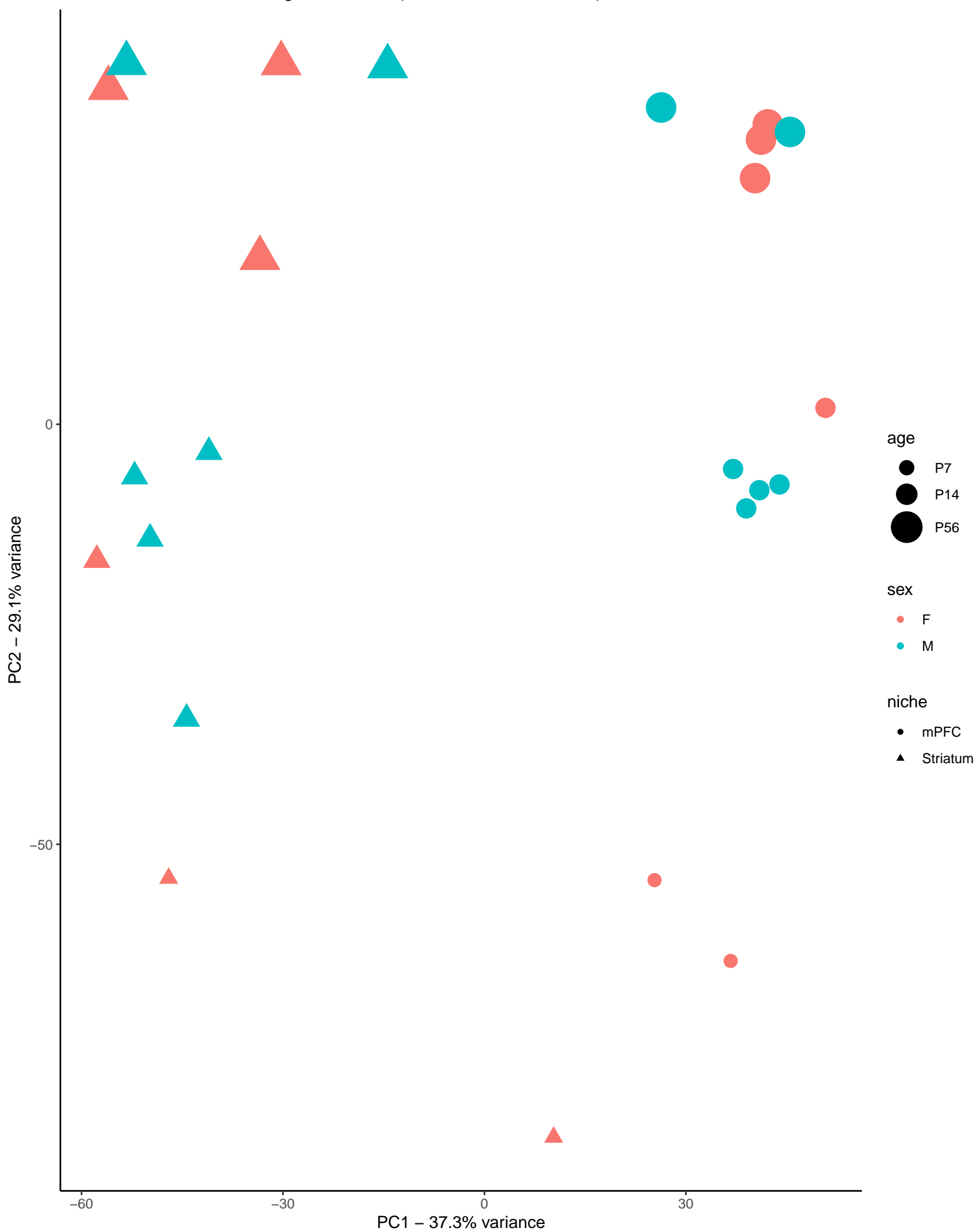

Supplement: Supplementary file 3 [file Image_3.PDF]

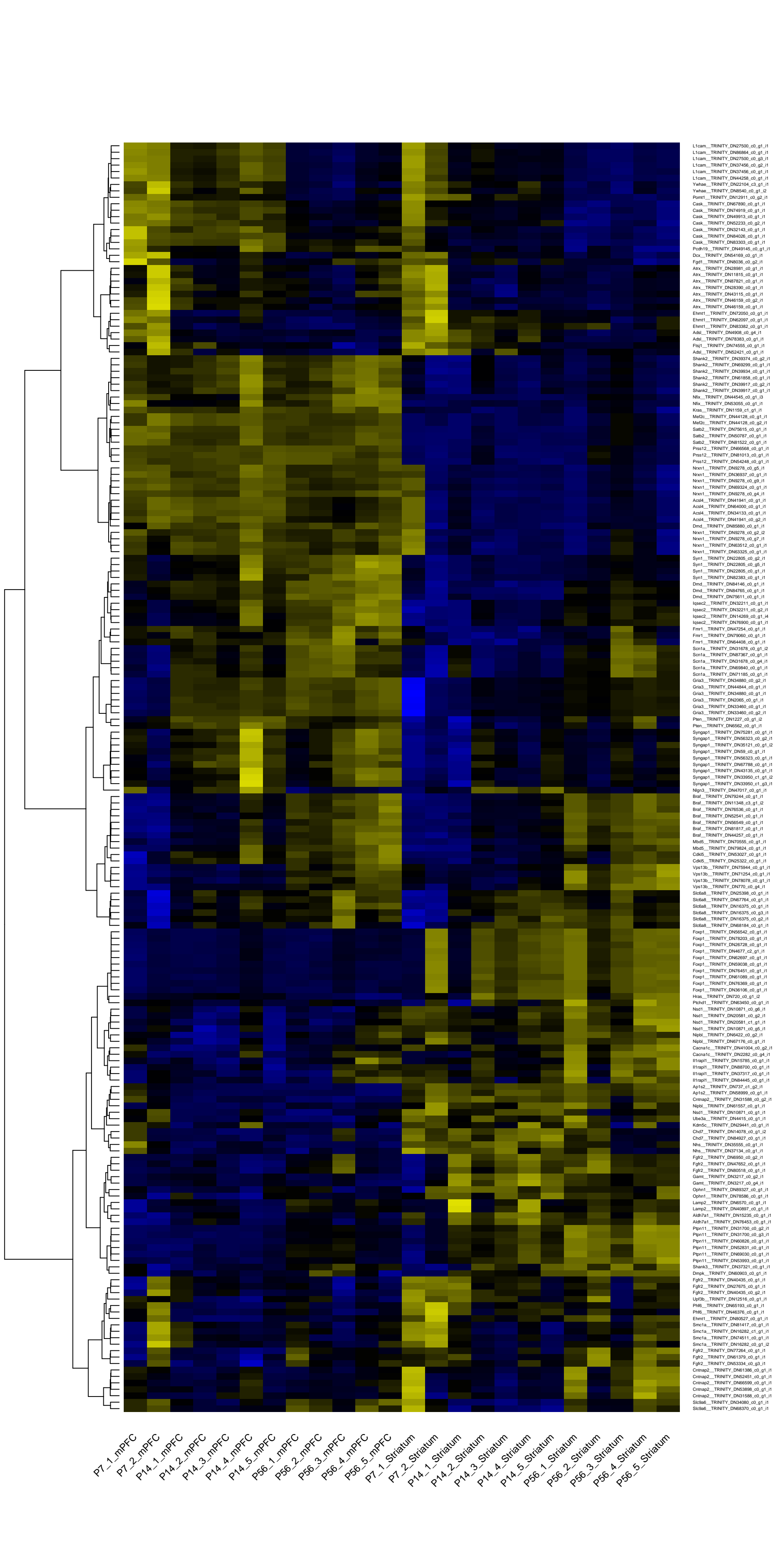

Supplement: Supplementary file 5 [file Image_5.PDF]
