# Supplementary material for: Identification of Natural Antisense Transcripts in Mouse Brain and Their Association With Autism Spectrum Disorder Risk Genes
Source: Front Mol Neurosci. 2021 Feb 25;14:624881. doi: 10.3389/fnmol.2021.624881 (PMC7947803; doi:10.3389/fnmol.2021.624881)
Supplement: Supplementary file 4 [file Image_4.PDF]

No	Antisense ID
1	Mef2c_F_65
2	Crebbp_R_1
3	Rpgrip1l_R_37
4	Foxp1_R_28
5	Cntnap2_F_13
6	Prss12_F_10
7	Syngap1_F_4
8	Syngap1_F_9
9	Cacna1c_R_15
+	18S

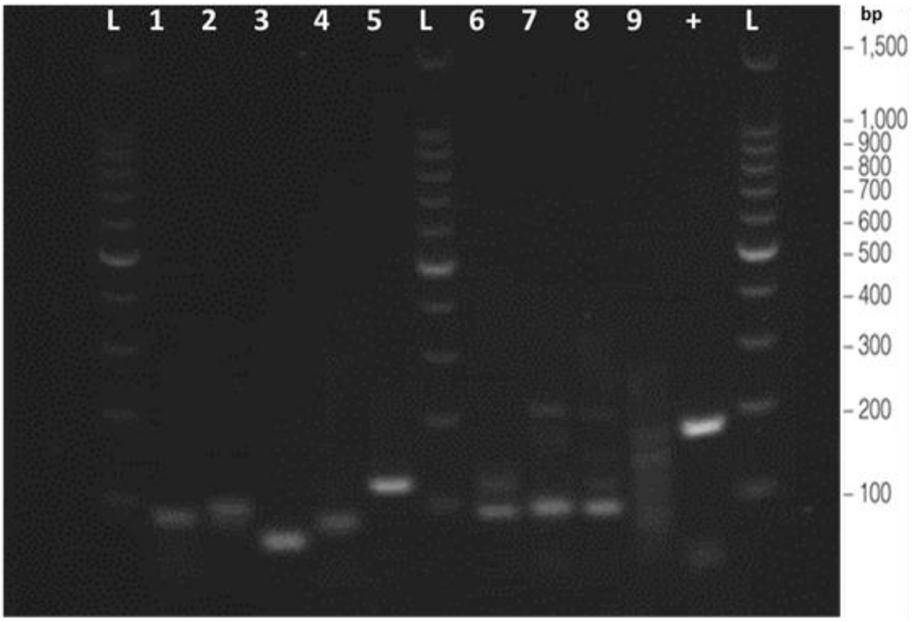

Figure S4. Verification of Selected *De Novo* Contigs as Natural Antisense Transcripts

9 antisense *de novo* contigs were verified using custom made TaqMan Probes in PCR. 18S transcript was used as a positive control. + is positive control. Agarose gel electrophoresis of PCR products. All PCR products of antisense transcripts, except for Cacna1c_R_15 (9), separated to expected fragment size. L is DNA ladder, bp is base pair.
