# Supplementary material for: Identification of Natural Antisense Transcripts in Mouse Brain and Their Association With Autism Spectrum Disorder Risk Genes
Source: Front Mol Neurosci. 2021 Feb 25;14:624881. doi: 10.3389/fnmol.2021.624881 (PMC7947803; doi:10.3389/fnmol.2021.624881)
Supplement: Supplementary file 6 [file Image_6.PDF]

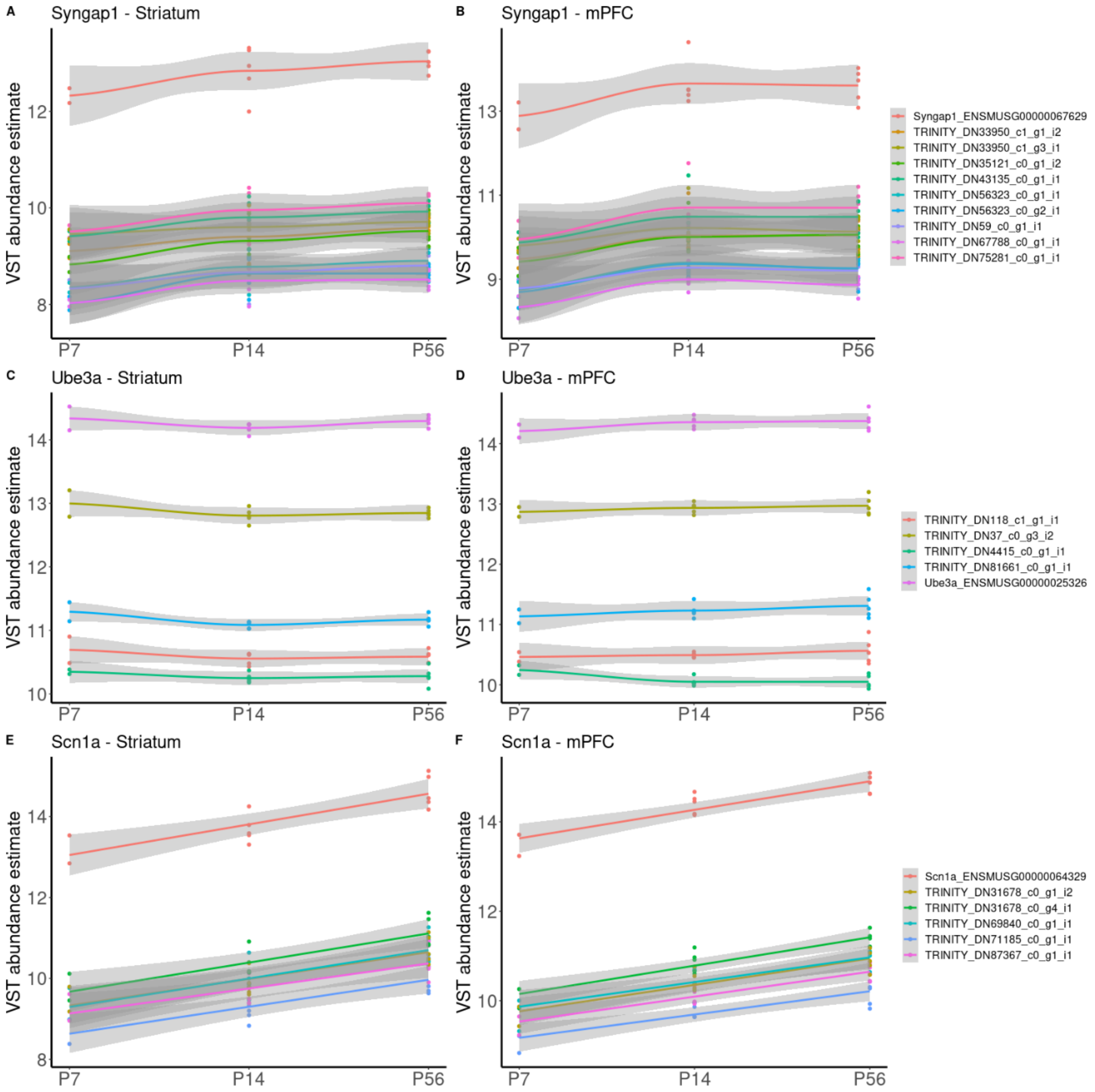

Figure S6. Correlation of Selected ASD Genes and Their Antisense Contig Partners through Development.VST abundance estimate for *Syngap1* in Striatum **(A)** and mPFC **(B)** and its antisense partners (TRINITY contigs). VST abundance estimate for *Ube3a* in Striatum **(C)** and mPFC **(D)** and its antisense partners (TRINITY contigs). VST abundance estimate for *Scn1a* in Striatum **(E)** and mPFC **(F)** and its antisense partners (TRINITY contigs). VST: variance stabilizing transformation. A linear model did not accurately fit the *Syngap1* and *Ube3a* expression dynamics, thus a Loess regression is shown for these genes. The regression for *Scn1a* is a linear model. Shaded areas indicate 95% confidence intervals.
