# Supplementary material for: Identification of Natural Antisense Transcripts in Mouse Brain and Their Association With Autism Spectrum Disorder Risk Genes
Source: Front Mol Neurosci. 2021 Feb 25;14:624881. doi: 10.3389/fnmol.2021.624881 (PMC7947803; doi:10.3389/fnmol.2021.624881)
Supplement: Supplementary file 15 [file Table_1.DOCX]

**Table S1. Biological Sex of the Animals**

| **Animal ID** | **Biological Sex** |
| --- | --- |
| P7-1 | Female |
| P7-2 | Female |
| P14-1 | Male |
| P14-2 | Male |
| P14-3 | Male |
| P14-4 | Female |
| P14-5 | Male |
| P56-1 | Female |
| P56-2 | Female |
| P56-3 | Male |
| P56-4 | Female |
| P56-5 | Male |
