# Supplementary material for: Identification of Natural Antisense Transcripts in Mouse Brain and Their Association With Autism Spectrum Disorder Risk Genes
Source: Front Mol Neurosci. 2021 Feb 25;14:624881. doi: 10.3389/fnmol.2021.624881 (PMC7947803; doi:10.3389/fnmol.2021.624881)
Supplement: Supplementary file 16 [file Table_2.DOCX]

**Table S2. List of Differentially Expressed Genes Between Sexes**

| ENSMUSG00000027833 Shox2 |
| --- |
| ENSMUSG00000035150 Eif2s3x |
| ENSMUSG00000035299 Mid1 |
| ENSMUSG00000036913 Trim67 |
| ENSMUSG00000041596 Nlrp5-ps |
| ENSMUSG00000042812 Foxf1 |
| ENSMUSG00000056673 Kdm5d |
| ENSMUSG00000056987 Fam71d |
| ENSMUSG00000068457 Uty |
| ENSMUSG00000069045 Ddx3y |
| ENSMUSG00000069049 Eif2s3y |
| ENSMUSG00000069053 Uba1y |
| ENSMUSG00000072940 Gm10443 |
| ENSMUSG00000073125 Xlr3b |
| ENSMUSG00000079297 Gm2223 |
| ENSMUSG00000086265 Marcksl1-ps4 |
| ENSMUSG00000095134 Mid1-ps1 |
| ENSMUSG00000098743 Gm27927 |
| ENSMUSG00000099312 Gm27733 |
| ENSMUSG00000099809 Gm18665 |
| ENSMUSG00000101059 Gm4017 |
